# Effect of carbonate apatite nanoparticles on the physicochemical properties and cytocompatibility of an experimental dental adhesive

**DOI:** 10.2340/biid.v13.45433

**Published:** 2026-02-11

**Authors:** Heba E. AbdelRazik, Jon E. Dahl, Simen E. Kopperud

**Affiliations:** a Department of Orthopaedics and Traumatology, Faculty of Medicine, University of Turku, Turku, Finland; b Nordic Institute of Dental Materials (NIOM), Oslo, Norway; c Department of Paediatric Dentistry and Oral Health Psychology, Faculty of Dentistry, University of Oslo, Oslo, Norway

**Keywords:** Physicochemical properties, degree of conversion, water sorption/solubility, cytotoxicity, nanofillers, carbonate apatite, silanization, surface treatment, experimental adhesive

## Abstract

**Objective:**

The study aimed to evaluate the effect of incorporating silanized and non-silanized carbonate apatite nanoparticles (CA5n) on the physicochemical properties and cytotoxicity of a hydrophilic experimental adhesive.

**Materials and methods:**

A dental adhesive composed of 70% bisphenol A diglycidyl ether dimethacrylate, 28.75% 2-hydroxyethyl methacrylate, 0.25% camphorquinone, and 1% ethyl N, N-dimethyl-4-aminobenzoate was used. CA5n, synthesized with a CO₃/PO₄ molar ratio of 5, was used in both silanized and non-silanized forms at concentrations of 0.5, 1, 2 or 4% (wt). The degree of conversion (DC) was measured using Fourier Transform Infrared spectroscopy (n = 5) immediately, 5 minutes, and 60 minutes after curing. Water sorption and solubility were evaluated over 28 days (n = 6). The cytotoxicity of 24-hour and 7-day extracts (n = 6) was assessed using 3-(4,5-dimethylthiazol-2-yl)-2,5-diphenyltetrazolium bromide (MTT) assay in triplicate. DC data were statistically analyzed using a mixed model. Independent-samples t-tests and Wilcoxon tests were used, depending on data normality, to analyze water sorption, solubility, and cell viability results. Statistical significance was set at p < 0.05.

**Results:**

The addition of CA5n significantly increased the DC over time, regardless of the concentration or silanization status. All CA5n groups exhibited high water sorption and acceptable water solubility, unaffected by concentration or silanization. Water sorption increased significantly with higher CA5n concentrations compared to the control, except in the 0.5% non-silanized (NS) group. Water solubility remained below 7.5 μg/mm³ (ISO 4049 requirement), with no significant differences among groups. Cell viability decreased significantly across all experimental groups compared to the negative control, with similar effects observed in both 24-hour and 7-day extracts.

**Conclusion:**

Adding CA5n to an experimental adhesive increased the DC and the water sorption. According to ISO 10993-5, the experimental adhesive, with and without CA5n, demonstrated low cytotoxicity potential.

**KEY MESSAGES**

- Adding CA5n to the experimental dental adhesive increased the degree of conversion and water sorption.
- Incorporation of CA5n maintained a low cytotoxicity.
- Silanization of CA5n did not significantly change the results compared to those of non-silanized CA5n.

## Introduction

Dentin adhesion has long been challenging due to the complex composition of dentin, the size of the adhesion area, the depth of remaining dentin, enzymatic activity, and other factors [1]. Several factors influence the longevity of dental composite restorations, including the formulation of the adhesive materials and the bonding procedures employed [1]. Continuous advances in adhesive chemistry and application techniques have improved the simplicity and versatility of dental adhesives [2, 3]. However, achieving a durable and stable bond remains difficult, as the adhesive interface is continuously exposed to moisture, mechanical stress, and enzymatic degradation in the oral cavity [2, 4].

For long-lasting restorations, physicochemical properties such as the degree of conversion (DC), water sorption, and water solubility are relevant to achieving adequate bond strength [5]. Insufficient DC reduces polymer crosslinking, leading to weaker bond strength and increased residual monomer release, while excessive water sorption and solubility accelerate the hydrolytic degradation of the resin matrix [6, 7]. Hydrophobic adhesives typically have a limited ability to penetrate moist dentin, whereas hydrophilic formulations, although compatible with the wet substrate, tend to absorb more water on aging, accelerating plasticization and degradation [8]. To address these limitations, different strategies have been proposed, such as optimizing filler composition [8], modifying bonding protocols [9, 10], and altering curing methods [11].

A variety of inorganic nanofillers, including hydroxyapatite [12], bioactive glass [13], calcium phosphate derivatives [14], and zinc compounds [15], have been explored to enhance the performance of dental adhesives [16]. Among calcium phosphate-based materials (CaP), carbonate apatite has garnered particular interest due to its chemical and structural similarity to natural hard tissues. Carbonate apatite is a carbonate-substituted form of hydroxyapatite that more closely resembles the mineral component of enamel, dentin, and bone [17]. Previous studies have shown that bioactive carbonate apatite cements exhibit higher compressive strength than traditional calcium phosphate cements and can stimulate dentin-pulp regeneration through ion release [18], evolving signaling pathways such as Mitogen-Activated Protein Kinase (MAPK), Wingless-related integration site / Beta-catenin (Wnt/β-catenin), and Transforming Growth Factor-beta / Smad (TGF-β/Smad) [19–21].

Nanotechnology has made tremendous progress across many fields, which has promoted its use in medicine and dentistry [22]. The growing applications of nanostructured materials and their unique characteristics encourage researchers to deepen their exploration and improve their performance [23]. In the present study, nanoparticles made from carbonate apatite synthesized with a CO_3_/PO_4_ molar ratio of 5 (CA5n) were investigated as filler particles in an experimental dental adhesive. Due to biomimetic composition and nanoscale surface area, CA5n may enhance filler dispersion, improve polymer-filler interaction, and release calcium and phosphate ions that support mineral deposition and tissue compatibility [24]. Despite this potential, no data seem to exist in the literature on the effect of adding carbonate apatite nanoparticles to dental adhesives.

Evaluating the biological response to these materials is equally important to ensure their safety and compatibility with oral tissues. According to the ISO 10993-5:2009 standard for the biological evaluation of medical and dental materials, L-929 mouse fibroblast cells are the recommended in vitro model for assessing cytotoxicity [25]. The study aimed to evaluate the effect of incorporating silanized and non-silanized CA5n at concentrations of 0.5, 1, 2, and 4 (wt%) on the physicochemical properties and cytotoxicity of a hydrophilic experimental adhesive. The null hypothesis was that there was no difference in (1) DC, (2) water sorption and solubility, and (3) cytotoxicity between the experimental adhesive with and without silanized or non-silanized CA5n.

## Materials and methods

### Nanoparticles preparation and characterization

**Figure 1:**
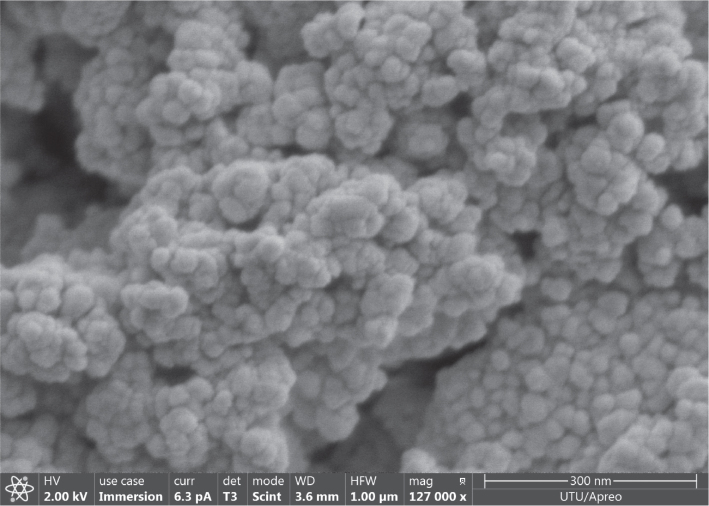
SEM image of synthesized carbonate apatite nanoparticles demonstrating sphere-shaped agglomerated nanoparticles of size less than < 100 nm. Magnification: 127,000X. SEM: scanning electron microscope.

Carbonate apatite microparticle powder previously synthesized with a CO_3_/PO_4_ molar ratio of 5 was used to prepare carbonate apatite nanoparticles (CA5n) [26]. The microparticle powder was wet milled in a milling machine (Planetary Micro Mill, Pulverisette 7, Fritsch, Idar-Oberstein, Germany) at 300 rpm for 10 minutes, paused for 4 minutes, and the cycle was repeated 55 times. After that, the particles were collected in 15 mL centrifuge tubes, sonicated, and then centrifuged; the resulting pellet was collected. For characterization of CA5n, a Zetasizer Nano ZS (Malvern Instruments, Malvern, UK), an X-ray diffraction (XRD) instrument (Malvern Panalytical Empyrean, Malvern, UK), and a scanning electron microscope (SEM) (FEI/Thermo Scientific Inc., Hillsboro, Oregon, USA) were used (Figure 1).

Silanization of the nanoparticles was performed by preparing a silane mixture containing 4% by volume silane coupling agent (3-methacryloyloxypropyltrimethoxysilane) (Sigma-Aldrich, St. Louis, MO, USA) in 96% of 90% ethanol (≥ 99.45% EtOH, Sigma-Aldrich, St. Louis, MO, USA) [27, 28]. CA5n particles were added to the silane mixture and left for 90 minutes in the dark. The mixture was then washed twice with Elix water, centrifuged, and the silanized CA5n particles were collected. The collected nanoparticles were dried in an oven (Termaks Model TS8024, Bergen, Norway) preset to 50°C, then heated to 130°C for 15–20 minutes, and subsequently maintained at 130°C for an additional 15 minutes. The dried CA5n particles were cooled in a desiccator and then immediately added to the experimental adhesive in different concentrations.

### Adding nanoparticles to the experimental adhesive

The experimental hydrophilic dental adhesive (Bisco Dental Products Co., Schaumburg, USA) was composed of 70% bis-GMA (bisphenol A diglycidyl ether dimethacrylate), 28.75% HEMA (2-hydroxyethyl methacrylate), 0.25% CQ (camphorquinone), and 1% EDMAB (ethyl N, N-dimethyl-4-aminobenzoate) [29]. Silanized and non-silanized CA5n were added at different percentages (0.5, 1, 2, 4% wt.) to the experimental adhesive. The CA5n containing adhesive was vortexed until the particles were well dispersed, then sonicated using a needle sonicator for 1 minutes (5 s on/2 s off, 30% amplitude) (Sonics Vibra-cell, Connecticut, USA) each time before use to prevent agglomeration and ensure homogeneity. The experimental dental adhesive without CA5n served as a control. The experimental setup is summarized in Table 1.

**Table 1:** Experimental groups and the tests performed in this study.

Groups	Silanization	DC	Ws & Wsl	Cell viability
0% (Control)	________	Immediately after curing		
0.5% NS	No			
0.5% Sil	Yes	5 minutes after curing		
1% NS	No		28 days	24-hour extracts
1% Sil	Yes			7-days extracts
2% NS	No	60 minutes after curing		
2% Sil	Yes			
4% NS	No			
4% Sil	Yes	(*n* = 5/group)	(*n* = 6/group)	(*n* = 6/group)

CA5n nanoparticles were added to the experimental adhesive at concentrations of 0.5, 1, 2, and 4% wt. DC: degree of conversion, Ws & Wsl: water sorption and water solubility, NS: non-silanized particles, Sil: silanized particles.

### Degree of conversion

Test specimens (*n* = 5/group according to previous study [30]) were prepared using a circumferential stainless-steel mold with a thickness of 0.6 mm and a diameter of 6 mm. The adhesive was placed into the mold, immediately covered with a Mylar strip to prevent the formation of an oxygen-inhibition layer [31], and cured for 20 s using an LED light curing unit (Demi Ultra, Kerr, California, USA, irradiance 1767 mW/cm^2^ ± 5%) in continuous mode, resulting in a radiant exposure of 35.3 J/cm². Fourier Transform Infrared Spectroscopy (Varian model 670 FTIR, Agilent Technologies, California, USA), equipped with a universal attenuated total reflectance (ATR) accessory and baseline method, was utilized [32]. Post-curing measurements were taken at three time points: immediately, at 5 minutes, and at 60 minutes [29]. The ratio between the C = C aliphatic absorbance peaks at 1637 cm^-1^ and the C-C aromatic absorbance peaks at 1607 cm^-1^ was calculated to determine the DC according to the following equation [33]:


$$\text{DC }(\%)=1-\frac{R(cured)}{R(uncured)}X100$$

where ‘R’ is the ratio between the aliphatic and aromatic peaks at 1637 and 1607 cm^-1^, respectively.

### Water sorption and solubility

The specimens were prepared following ISO 4049 specification [34], except for their dimensions [35]. Water sorption was monitored for 28 days, along with the water solubility of the material. Discs (*n* = 6/group) of experimental adhesive incorporated with 0.5, 1, 2, and 4% wt. nanoparticles of carbonate apatite (CA5n) with and without silanization were prepared with a diameter of 6 ± 0.1 mm and a thickness of 0.5 ± 0.02 mm, verified by a digital caliper (Mitutoyo Digimatic Caliper Europe GmbH, Neuss, Germany) [36]. Discs of experimental adhesive without CA5n served as controls. The discs were kept in a desiccator at 37°C and weighed daily using an analytical balance (Mettler Toledo Balance XS205DU, Greifensee, Switzerland) until a constant mass (*m1*) was reached, with a variation of less than 0.2 mg within 24 hours. Afterwards, the specimens were immersed in water and weighed daily for up to 7 days, and then again at 14 days and 28 days (*m2*)*.* After 28 days in water, the specimens were transferred to new dry vials and stored in a 37°C incubator. They were weighed daily until they reached a stable weight (*m3*). Water sorption (Ws) and water solubility (Wsl) were calculated using the following equations [37]:


$$\text{Ws}=\frac{m2-m3}{V},\;\;\text{Ws}1=\frac{m1-m3}{V}$$

where ‘V’ is the sample volume in mm^3^.

### Cell viability

The L-929 mouse fibroblast cell line and the 3-(4,5-dimethylthiazol-2-yl)-2,5-diphenyltetrazolium bromide assay (MTT assay), as specified in ISO 10993-5:2009 [25], were used to evaluate cytotoxicity following the incorporation of CA5n with and without silanization into the experimental dental adhesive. Discs (*n* = 6/group) were prepared as described for the water sorption and solubility test method. The prepared discs were stored individually in glass containers filled with 500 μL of cell medium. The surface area-to-volume ratio was 1.4 cm^²^/mL, within the range of 0.5–6 cm²/mL, as specified by ISO standard [25]. The sealed extract bottles were placed in a water bath (Julabo, Göteborg, Sweden) at 37°C for 24 hours and 7 days. L-929 cells, from the third passage, were used in triplicate, seeded at a cell density of 1 × 10^4^ cells/well in 96-well plates and incubated at 37°C, 5% CO_2_, and 95% humidity in an incubator (Sanyo Incubator, Tokyo, Japan) for 24 hours [38]. A positive control of 2-hydroxyethylmethacrylate (HEMA, Fluka Chemie AG, Buchs, Switzerland) (10 mM), a negative control with cell medium and cells, and blanks with only the cell medium were used in this study. The absorbance was measured at 570 nm using a spectrophotometer (Synergi H1, Biotek, Vermont, USA) [35, 38]. The L-929 cell line was purchased from the European Collection of Authenticated Cell Cultures (ECACC, Public Health England) and tested for mycoplasma using the MycoAlert Mycoplasma Detection kit (Lonza, Rockland, ME, USA).

### Statistical analysis

A mixed model, a specific type of ANOVA (Stata version 17.0, College Station, TX, USA), was used to evaluate the DC results, with CA5n concentration and silanization as fixed factors and time as a repeated (within-specimen) factor. Concentration, silanization, and time were considered categorical variables. The Shapiro-Wilk test was used to assess the normality of the water sorption, solubility, and cell viability data. Based on the results of the normality test, the independent-samples *t*-test was applied to normally distributed data, whereas the Wilcoxon rank-sum test was used for data that did not meet the normality assumption (R version 4.4.1, Vienna, Austria). Statistical significance was set at *p* < 0.05.

## Results

The SEM image (Figure 1) shows agglomerated CA5n nanoparticles with irregular to nearly spherical shapes. The particles’ size was below 100 nm, confirming the successful synthesis of nanoscale particles. The results of the DC measurements are listed in Table 2. Adding CA5n to the adhesive increased the DC, and the effect was independent of concentration and silanization of the nanoparticles. Time significantly increased the DC, with values at both 5 minutes and 60 minutes being significantly higher than immediately after curing in all groups (*p* < 0.001).

**Table 2:** Degree of conversion (%) mean ± standard deviation.

Groups (CA5n)	Immediately after curing	5 minutes after curing	60 minutes after curing
0%	65.2 ± 0.4^Aa^	68.2 ± 0.7^Bc^	72.2 ± 1.1^Ce^
0.5% NS	67.8 ± 0.6^Db^	70.7 ± 0.5^Ed^	74.6 ± 0.5^Ff^
0.5% Sil	67.9 ± 0.5^Db^	70.5 ± 0.5^Ed^	74.5 ± 0.2^Ff^
1% NS	67.6 ± 0.3^Db^	70.2 ± 0.6^Ed^	74.6 ± 0.3^Ff^
1% Sil	68.1 ± 0.8^Db^	70.8 ± 0.5^Ed^	74.9 ± 0.7^Ff^
2% NS	66.8 ± 2.2^Db^	70.4 ± 0.2^Ed^	74.7 ± 0.2^Ff^
2% Sil	67.1 ± 0.7^Db^	69.9 ± 0.7^Ed^	74.1 ± 0.6^Ff^
4% NS	67.4 ± 0.8^Db^	70.3 ± 0.6^Ed^	74.5 ± 0.5^Ff^
4% Sil	68.1 ± 0.4^Db^	70.9 ± 0.4^Ed^	75.1 ± 0.3^Ff^

Different concentrations of CA5n (0.5, 1, 2, and 4% wt) added to the experimental adhesive and the control (0%). NS: non-silanized particles, Sil: silanized particles. Different superscript upper-case letters show statistically significant differences in each row and different superscript lower-case letters show statistically significant differences in each column. Statistical significance was set at *p* < 0.05.

Water sorption and water solubility after 28 days are shown in Table 3. All CA5n groups showed significantly higher water sorption than the control group (*p* < 0.05), except for the 0.5% NS group (*p* = 0.064). All CA5n-containing groups exhibited acceptable water solubility, as defined by ISO 4049 specification [34] (<7.5 μg/mm³). There were no significant differences among the groups (*p* > 0.05).

**Table 3:** Water sorption after 28 days (D28), and water solubility.

Groups (CA5n)	D28	Water solubility
0%	80.57 ± 3.53^a^	4.42 ± 1.21
0.5% NS	84.53 ± 2.26^a^	3.15 ± 3.09
0.5% Sil	87.33 ± 2.35^b^	2.32 ± 1.95
1% NS	85.69 ± 3.46^b^	3.92 ± 2.1
1% Sil	87.05 ± 2.06^b^	3.78 ± 1.57
2% NS	88.60 ± 1.99^b^	3.91 ± 1.68
2% Sil	84.55 ± 2.25^b^	4.13 ± 3.05
4% NS	88.49 ± 2.47^b^	5.52 ± 3.08
4% Sil	85.78 ± 2.35^b^	5.58 ± 3.12

Mean ± standard deviation in μg/mm^3^. Different concentrations of CA5n (0.5, 1, 2, and 4% wt.) added to the experimental adhesive and the control (0%). NS: non-silanized particles, Sil: silanized particles. Different superscript small letters indicate statistically significant difference for water sorption at D28 between different groups compared to the control group. Statistical significance was set at *p* < 0.05.

**Figure 2:**
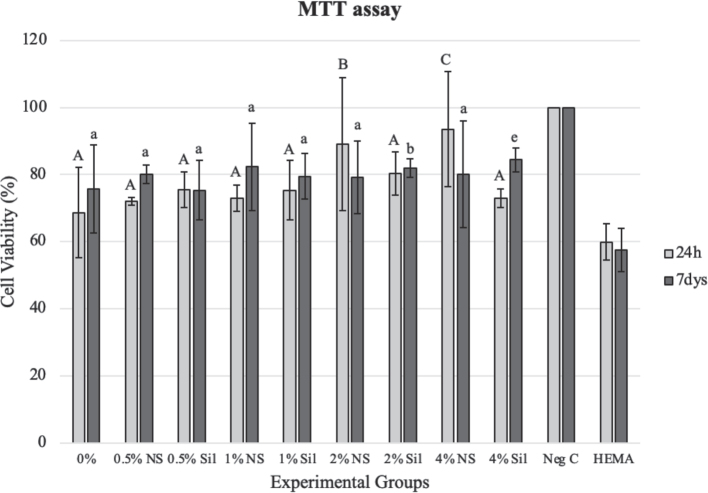
Effects of varying concentrations of CA5n added to the experimental adhesive on cell viability for 24-hour and 7-day extracts. Cell viability data are expressed as a percentage of the negative control cultures. NS: non-silanized particles; Sil: silanized particles. Different uppercase letters indicate statistical significance between different CA5n-containing groups and the control group (experimental adhesive without CA5n) at 24 hours (*p* < 0.05). Different lowercase letters indicate statistical significance between CA5n-containing groups and the control group (experimental adhesive without CA5n) at 7 days (*p* < 0.05).

MTT assay results are shown in Figure 2. Extracts after 24 hours and 7 days from all experimental groups and the control group showed significantly lower cell viability compared to the negative control (cell culture only) (*p* < 0.05). After 24 hours, cell viability was significantly higher in the 2% NS (*p* = 0.039) and 4% NS (*p* = 0.004) groups compared to the experimental adhesive without CA5n. In the silanized groups, particle concentration did not significantly influence cytotoxicity compared to the experimental adhesive without CA5n (*p* > 0.05). After 7 days, there were no significant differences in cytotoxicity among the adhesive groups (*p* > 0.05), except for the 2% Sil (*p* = 0.029) and 4% Sil (*p* = 0.009) groups, which exhibited significantly lower cytotoxicity than the other groups. Similar effects were observed in both 24 hours and 7-day extracts.

## Discussion

This study investigated the effect of incorporating carbonate apatite nanoparticles (CA5n) into a hydrophilic experimental dental adhesive on its physicochemical and biological properties. The results showed that adding CA5n increased the DC and the water sorption. Water solubility results met ISO 4049 standard [34] (<7.5 μg/mm^3^). Additionally, all formulations demonstrated acceptable cytotoxicity with L929 fibroblasts, as per ISO 10993-5 standard [25]. Silanizing the nanoparticles did not significantly change the results compared to those of non-silanized CA5n.

The DC reflects the efficiency of resin polymerization and is a critical determinant of mechanical strength, water resistance, and biocompatibility [3]. Typically, resin systems achieve conversion rates ranging from 52 to 75% [3]. Unreacted residual monomers may leach into the surrounding environment, eliciting toxic or irritating responses [3]. Previous studies have shown that adding inorganic fillers to dental adhesives can improve DC, likely by enhancing light scattering, reducing polymerization shrinkage, or modifying monomer-filler interactions [3]. However, other investigations reported no effect [39, 40] or even a detrimental impact [41, 42] of fillers on DC, possibly due to filler agglomeration, reduced monomer mobility [43, 44], mismatched refractive indices [45, 46], or increased viscosity [47]. It is therefore important to limit filler loading; in this study, the CA5n concentration was capped at 4% wt. Incorporating CA5n increased the DC to approximately 75%.

Silanization is often reported as an effective strategy to enhance filler-matrix adhesion and improve the mechanical and biological performance of resin composites by promoting covalent bonding between the inorganic filler surface and the organic resin matrix [27]. However, other studies have shown inconsistent or negligible effects, likely due to differences in formulation or incomplete silane coupling [48, 49]. Moreover, because silane molecules are relatively hydrophobic, excessive surface modification may compromise resistance to hydrolytic degradation [50]. In the current study, there was no significant difference in DC between the two surface treatments. This suggests that, under the highly hydrophilic conditions of the tested adhesive system, polymerization efficiency was predominantly controlled by monomer composition and water/solvent content, rather than by silane-mediated interfacial effects.

Two important parameters, water sorption and solubility, were assessed in this study as indicators of adhesive quality [51]. Water sorption reflects the diffusion of moisture into the polymer matrix, which can chemically degrade the material, weaken the bond between the organic matrix and inorganic fillers, and promote the leaching of residual monomers [5]. All tested groups, including the control, exhibited higher water sorption values than the ISO 4049 limit of 40 μg/mm³. However, silanization did not appear to significantly affect water sorption. The high water sorption and solubility may be attributed to solubility occurring primarily within the resin matrix [50], the high surface area of CA5n, which can increase the filler-matrix interfacial region and facilitate water diffusion, and the resin formulation’s intrinsic hydrophilicity [52, 53].

Cytotoxicity analysis was performed to assess the biological behavior of the modified adhesive [54]. The MTT assay was used as a verified colorimetric assay that measures the influence of the extract of the tested material on the metabolic activity of a cell culture [55]. The release of unreacted monomers into the extraction medium may influence the cytotoxic potential of a resin-based test material. Monomer elution typically peaks within the first 24 hours after curing, but can continue for several days [56]. Although most leaching is reportedly limited to this initial period, some studies have found increased cytotoxic effects after 72 hours of cell exposure, indicating ongoing monomer diffusion from the resin matrix [55, 57]. The extent of elution depends on factors such as monomer composition, solvent type, and the degree of polymerization achieved during curing [58, 59].

The observed cell viability was approximately 70% of that in the negative control group across all tested groups (experimental adhesive with and without CA5n). The results indicate that the adhesive’s modification did not adversely affect its cytotoxicity, which could be rated low according to ISO 10993-5:2009 [25]. Extracts from resin-based materials mainly contain monomers and other unreacted chemicals [60]. Filler particles are most likely absent from the extract, which explains why adding CA5n particles did not potentiate the cytotoxicity of the experimental dental adhesive. The lower cytotoxicity observed in some CA5n-containing groups may be attributed to reduced resin matrix volume, improved DC, or unreacted monomers being adsorbed by filler particles [61–63].

This study was limited to evaluating the physicochemical properties and cytotoxicity of experimental dental adhesives containing CA5n. Further research is needed to assess the remineralization potential of these materials at the resin-dentin interface and to investigate the cellular signaling pathways underlying their biological interactions. Such studies would offer a more comprehensive understanding of the bioactivity and long-term performance of these adhesive systems.

## Conclusion

The addition of CA5n increased the DC of the experimental adhesive, indicating that the nanoparticles improved the polymerization process. The high water sorption and solubility found may be due to the adhesive’s inherent hydrophilicity rather than the CA5n filler itself. The low cytotoxic potential observed in the extracts was related to the experimental adhesive rather than adding CA5 nanoparticles. Silanization of CA5n did not significantly alter the evaluated properties compared to those of the non-silanized CA5n.

## Data Availability

The data that support this study’s findings are available upon reasonable request.
